# Pyrogallol-Phloroglucinol-6,6-Bieckolon Attenuates Vascular Smooth Muscle Cell Proliferation and Phenotype Switching in Hyperlipidemia through Modulation of Chemokine Receptor 5

**DOI:** 10.3390/md18080393

**Published:** 2020-07-27

**Authors:** Seyeon Oh, Myeongjoo Son, Chul-Hyun Park, Ji Tae Jang, Kuk Hui Son, Kyunghee Byun

**Affiliations:** 1 Functional Cellular Networks Laboratory, Lee Gil Ya Cancer and Diabetes Institute, Gachon University, Incheon 21999, Korea; seyeon8965@gmail.com (S.O.); mjson@gachon.ac.kr (M.S.); 2 Department of Anatomy & Cell Biology, Graduate School of Medicine, Gachon University, Incheon 21936, Korea; 3 Department of Thoracic and Cardiovascular Surgery, Gachon University Gil Medical Center, Gachon University, Incheon 21565, Korea; cdgpch@gilhospital.com; 4 Aqua Green Technology Co., Ltd., Smart Bldg., Jeju Science Park, Cheomdan-ro, Jeju 63243, Korea; whiteyasi@gmail.com

**Keywords:** *Ecklonia cava*, pyrogallol-phloroglucinol-6,6′-bieckol, vascular smooth muscle cell proliferation, vascular smooth muscle cell phenotype switching, chemokine receptor 5

## Abstract

Hyperlipidemia induces vascular smooth muscle cell (VSMC) proliferation and phenotype switching from contractile to synthetic. This process is involved in arterial remodeling via the chemokine ligand 5 (CCL5)/chemokine receptor 5 (CCR5) pathway. Arterial remodeling is related to atherosclerosis or intimal hyperplasia. The purpose of this study was to evaluate whether pyrogallol-phloroglucinol-6,6-bieckol (PPB) from *E. cava* reduces VSMC proliferation and phenotype switching via the CCL5/CCR5 pathway. The CCL5/CCR5 expression, VSMC proliferation and phenotypic alterations were evaluated using a cell model of VSMC exposed in hyperlipidemia, and an animal model of mice fed a high-fat-diet (HFD). The expression of CCL5/CCR5 increased in both the cell and animal models of hyperlipidemia. Treatment with PPB decreased CCL5/CCR5 expression in both models. The expression of contractile markers of VSMCs, including alpha-smooth muscle actin (α-SMA), smooth muscle myosin heavy chain (SM-MHC), and smooth muscle protein 22 alpha (SM22α), were decreased by hyperlipidemia and restored after treatment with PPB. The silencing of CCR5 attenuated the effects of PPB treatment. VSMC proliferation and the intima-media thickness of the aortas, increased with HFD and decreased after treatment with PPB. The VSMC proliferation ratio and messenger ribonucleic acid (mRNA) expression of cell cycle regulatory factors increased in the in vitro model and were restored after treatment with PPB. PPB treatment reduced VSMC proliferation and phenotype switching induced by hyperlipidemia through inhibition of the CCL5/CCR5 pathway.

## 1. Introduction

Under normal homeostatic conditions with healthy vascular physiology, vascular smooth muscle cells (VSMCs) have low baseline levels of proliferation [1,2]. However, in pathophysiological conditions, such as atherosclerosis or restenosis, after interventional therapy or intimal hyperplasia, VSMC proliferation is increased [1,2,3,4,5]. In healthy vessels, fully differentiated VSMCs are spindle-shaped, have contractile properties, and have a low proliferation rate [6]. The vascular wall remodeling process, which is induced by atherosclerosis or neointimal hyperplasia, leads to VSMC dedifferentiation or phenotype switching [6,7]. Phenotype switching induces VSMCs to change from a physiological contractile phenotype to a pathophysiological synthetic phenotype [8,9]. The synthetic VSMCs have rhomboid or epithelioid shape and exhibit a high proliferation rate and high rate of extracellular matrix (ECM) production [6,7].

Chemokine receptor 5 (CCR5) is a member of the β-chemokine receptor family and is expressed on macrophages, T cells, endothelial cells, and VSMCs [10,11]. The chemokine ligand 5 (CCL5, also known as regulated upon activation, normal T cell expressed and aresumably secreted (RANTES)), a ligand of CCR5, has been implicated in the pathophysiology of atherosclerosis through the mediation of VSMC phenotype switching and macrophage recruitment [12]. CCL5 has been shown to increase vascular wall thickness and induce leukocyte recruitment to the site of arterial injury during vascular remodeling [13]. Inhibition of CCL5 binding to CCR5 by met-RANTES decreased the formation of atherosclerotic plaques in mice [14]. Furthermore, CCR5 deletion in apolipoprotein E (ApoE)-deficient mice resulted in decreased neointima formation after arterial wire injury [15]. These data suggest that the CCL5/CCR5 signaling pathway plays a critical role in the pathogenesis of atherosclerosis or neointima formation [16].

The expression of CCL5 and CCR5 in VSMCs treated palmitic acid (PA) was increased, and increased expression level of CCL5 or CCR5 induced VSMC proliferation and synthetic phenotype changes [17]. Expression levels of CCL5 and CCR5 in the aorta were increased in mice fed a high-fat diet (HFD) as compared to those fed a regular fat diet (NFD) [17]. Mice that were fed an HFD that had genetic deficiencies in the CCL5/CCR5 pathway showed attenuated VSMC proliferation and reduced phenotype switching from the contractile to synthetic phenotype [17]. Those reports suggested that CCR5 may be a potential target for the treatment of atherosclerotic diseases, especially those induced by hyperlipidemia [17].

*Ecklonia cava* (*E. cava*) is an edible marine brown alga that is one of the richest sources of phlorotannin in nature and has anti-inflammatory and antioxidant activities [18,19]. Pyrogallol-phloroglucinol-6,6-bieckol (PPB), which is one of the single compound of *E. cava*, contains 15 OH groups which showed anti-oxidant efficacy [20]. Previous study showed that PPB significantly inhibited monocyte migration and decreased macrophage differentiation to the inflammatory type [20]. PPB treatment has also been shown to reduce monocyte-associated endothelial cell death and reduce VSMC proliferation or migration, which is typically induced by inflammatory monocytes [20]. However, it remains unknown whether PPB reduces VSMC proliferation and phenotype switching through the CCL5/CCR5 pathway, without the engagement of monocytes or other immune cells, which increases inflammation.

The purpose of our study was to evaluate whether PPB attenuates CCL5/CCR5 expression induced by HFD and attenuates VSMC proliferation and reduced phenotype switching, which is a pathophysiological characteristic of atherosclerosis or intimal hyperplasia induced by HFD.

## 2. Results

### 2.1. PPB Reduced Expression of CCL5/CCR5 in VSMCs Treated With PA and in The Aortas of Mice Fed an HFD

**Figure 1:**
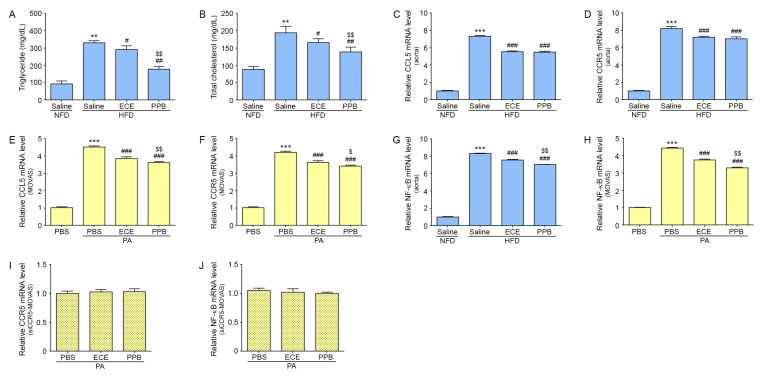
Effects of ECE (E. cava extract) and PPB on triglyceride levels, total cholesterol, and expression of CCL5, CCR5 and NF-κB. (**A**,**B**) The triglyceride and total cholesterol levels were measured using the serum of HFD (high-fat-diet) or NFD (regular fat dietfed mice). Triglyceride and total cholesterol levels were increased by HFD and were significantly decreased after treatment with ECE or PPB. (**C**,**D**) In aorta tissue, CCL5 (**C**) and CCR5 (**D**) mRNA levels were increased by HFD. Addition of ECE and PPB decreased the CCL5 and CCR5 mRNA levels. (**E**,**F**) In vascular smooth muscle cells (MOVAS), CCL5 (**E**) and CCR5 (**F**) mRNA levels were increased) by treatment with PA. Addition of ECE or PPB decreased CCL5 and CCR5 mRNA levels. (**G**,**H**) NF-κB mRNA levels in aorta (**G**) and MOVAS (**H**) were increased by HFD or treatment with PA. Addition of ECE or PPB decreased NF-κB mRNA levels. (**I**,**J**) In MOVAS upon silencing of the CCR5 gene, mRNA levels of CCR5 (**I**) and NF-κB (**J**) were unchanged in all groups. Data represent the means ± SD. ** *p* < 0.01, *** *p* < 0.001, vs. the NFD or PBS-treated group; # *p* < 0.05, ## *p* < 0.01, ### *p* < 0.001 vs. the HFD or PA-treated group; $ *p* < 0.05 and $$ *p* < 0.01 vs. HFD/ECE or PA/ECE group. CCL5, chemokine ligand 5; CCR5, chemokine receptor 5; ECE, extract of *Ecklonia cava*; HFD, high-fat diet group; NFD, normal fat diet group; NF-κB, nuclear factor kappa-light-chan-enhancer of activated B cells; mRNA, messenger ribonucleic acid; PA, palmitate acid; PBS, phosphate-buffered saline; PPB, pyrogallol-phloroglucinol-6,6-bieckol.

The serum levels of triglycerides and total cholesterol in HFD fed mice were higher than those in NFD fed mice. Mice fed an HFD treated with *E. cava* extract (ECE) or PPB had reduced levels of triglycerides and total cholesterol than mice fed an HFD (Figure 1A,B). The levels of triglyceride and total cholesterol of PPB treated group was significantly lower than those of ECE treated group.

The expression of both of CCL5 and CCR5 in the aortic tissue of mice fed an HFD were increased as compared to the expression in mice fed an NFD (Figure 1C,D). Expression of CCL5 and CCR5 was significantly decreased in mice treated with ECE or PPB. However, the levels of expression of CCL5 and CCR5 were not statistically significantly different between mice treated with ECE and mice treated with PPB (Figure 1C,D).

The expression of both of CCL5 and CCR5 the VSMCs (MOVAS) was increased after PA treatment and was decreased after treatment with either ECE or PPB (Figure 1E,F). The attenuation of CCL5/CCR5 expression was significantly greater in the PPB-treated MOVAS than those treated with ECE (Figure 1E,F).

The expression of nuclear factor kappa-light-chain-enhancer of activated B cells (NF-κB) in the aorta of HFD fed mice was significantly increased than that of NFD fed mice (Figure 1G). The expression of NF-κB in the aorta which was increased by HFD was decreased by treating either ECE or PPB. The decreasing effect was significantly greater by treating PPB than ECE.

The expression of NF-κB in MOVAS was increased by treating PA and that was significantly decreased by treating either ECE or PPB (Figure 1H). The decreasing of effect was significantly greater by treating PPB than ECE.

To confirm whether CCR5 is involved in VSMC phenotype switching, MOVAS were transfected with a CCR5-specific siRNA and treated with either ECE or PPB after PA treatment. CCR5 siRNA inhibit CCR5 protein expression by almost 90% at 100 nmol/L, as compared with cells transfected with control siRNA. The expression of CCR5 was not significantly decreased by treating with either ECE or PPB in CCR5-specific siRNA-transfected MOVAS (siCCR5-MOVAS) treated with PA (Figure 1I). The expression of NF-κB ws not significantly changed in PA treated siCCR5-MOVAS after treating either ECE or PPB (Figure 1J).

### 2.2. PPB Attenuated VSMC Phenotype Switching Induced by PA

**Figure 2:**
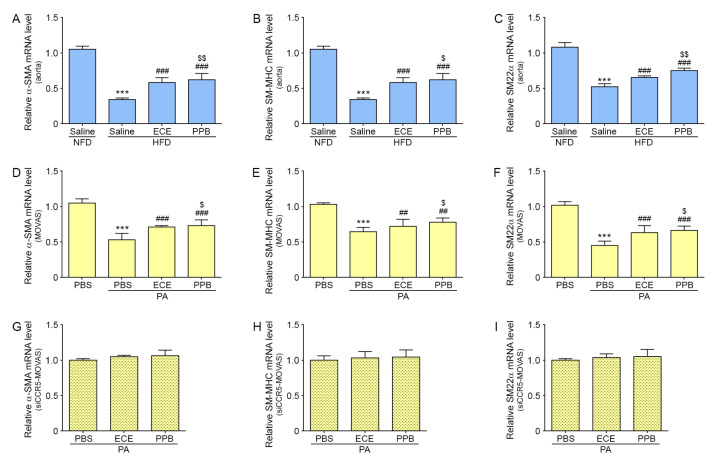
Effects of ECE and PPB on the phenotype change of smooth muscle cells. (**A**–**C**) In aorta tissue, the mRNA levels of the contractile phenotype marker of vascular smooth muscle cells, including α-SMA, SM-MHC, and SM22α, were significantly decreased by HFD and were significantly increased when HFD was supplemented with ECE or PPB. PPB had the most significant effects on decreasing the contractile phenotype induced by HFD. (**D**–**F**) In MOVAS cells, the mRNA levels of the contractile phenotype markers were significantly increased by treatment with PA and were significantly decreased when PA was supplemented with ECE or PPB. PPB had the most significant effects on decreasing the contractile phenotype induced by PA. (**G**–**I**) Upon silencing the CCR5 gene in MOVAS cells, the mRNA levels of the contractile phenotype marker were unchanged in all groups. *** *p* < 0.001, vs. the NFD or PBS group; ## *p* < 0.01, ### *p* < 0.001 vs. the HFD or PA group; $ *p* < 0.05 and $$ *p* < 0.01 vs. HFD/ECE or PA/ECE group. α-SMA, alpha-smooth muscle actin; CCR5,chemokine receptor 5; ECE, extract of *Ecklonia cava*; HFD, high-fat diet group; NFD, normal fat diet group; mRNA, messenger ribonucleic acid; PA, palmitate acid; PBS, phosphate-buffered saline; PPB, pyrogallol-phloroglucinol-6,6-bieckol; SM22α, smooth muscle protein 22 alpha; SM-MHC, smooth muscle myosin heavy chain.

The expression of contractile markers of VSMC, including alpha-smooth muscle actin (α-SMA), smooth muscle myosin heavy chain (SM-MHC), and smooth muscle protein 22 alpha (SM22α) [21,22], were decreased in the aorta of HFD fed mice compared to NFD fed mice (Figure 2A–C). Those expressions were significantly increased by either of ECE or PPB treating, and those expressions of aorta of PPB-treated mice was significantly higher than those of ECE-treated mice.

The expression of α-MSA, SM-MHC, and SM22α decreased after PA treatment in MOVAS (Figure 2D–F). However, after treatment with ECE or PPB in PA-treated MOVAS, the expression of these markers significantly increased. This increase was more significant in MOVAS treated with PPB than in those treated with ECE (Figure 2D–F).

The expression of α-SMA, SM-MHC, and SM22α were not significantly changed by treating with either ECE or PPB siCCR5-MOVAS treated with PA (Figure 2G–I).

### 2.3. PPB Reduced Proliferation of VSMCs Induced by PA or HFD

**Figure 3:**
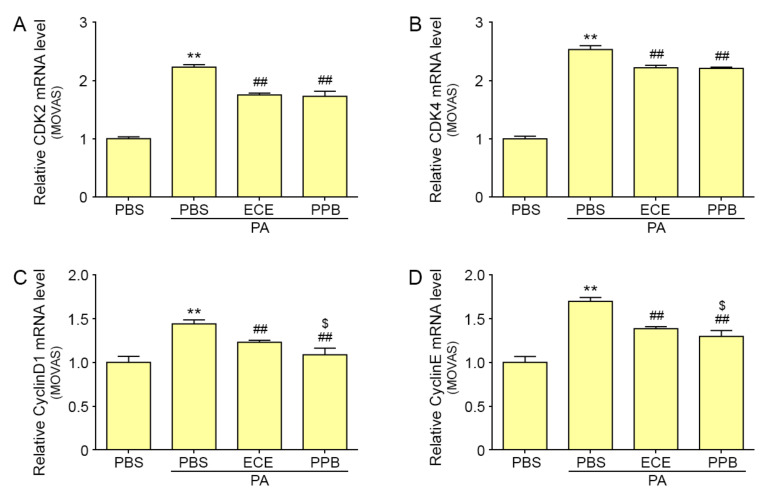
Effects of ECE and PPB on the expression of cell cycle-related mRNA levels. (**A**–**D**) In MOVAS cells, the cell cycle-related mRNA levels, including levels of CDK2, CDK4, Cyclin D1, and Cyclin E, were significantly increased after PA treatment and were significantly reduced when PA was supplemented with ECE or PPB. Treatment with PPB had the most significant effect on reducing the expression of cyclin D1 and cyclin E. Data represents means ± SD. ** *p* < 0.01, vs. the PBS group; ## *p* < 0.01 vs. the PA group; $ *p* < 0.05 vs. PA/ECE group. CDK, cyclin-dependent kinases; ECE, extracts of *Ecklonia cava*; PA, palmitate acid; PBS, phosphate-buffered saline; PPB, pyrogallol-phloroglucinol-6,6-bieckol.

The mRNA expression of cell cycle regulatory factors, including cyclin-dependent kinases (CDK) 2, CDK4, cyclin D1, and cyclin E, [23] were significantly increased by PA in MOVAS (Figure 3A–D). Those expressions were significantly decreased by treating with either ECE or PPB. The expression of CDK2 and CDK4 in PPB-treated MOVAS was not significantly lower than that in ECE-treated MOVAS (Figure 3A–B). However, the mRNA expression of cyclin D1 and cyclin E in PPB-treated MOVAS was significantly lower than that in ECE-treated MOVAS (Figure 3C–D).

**Figure 4:**
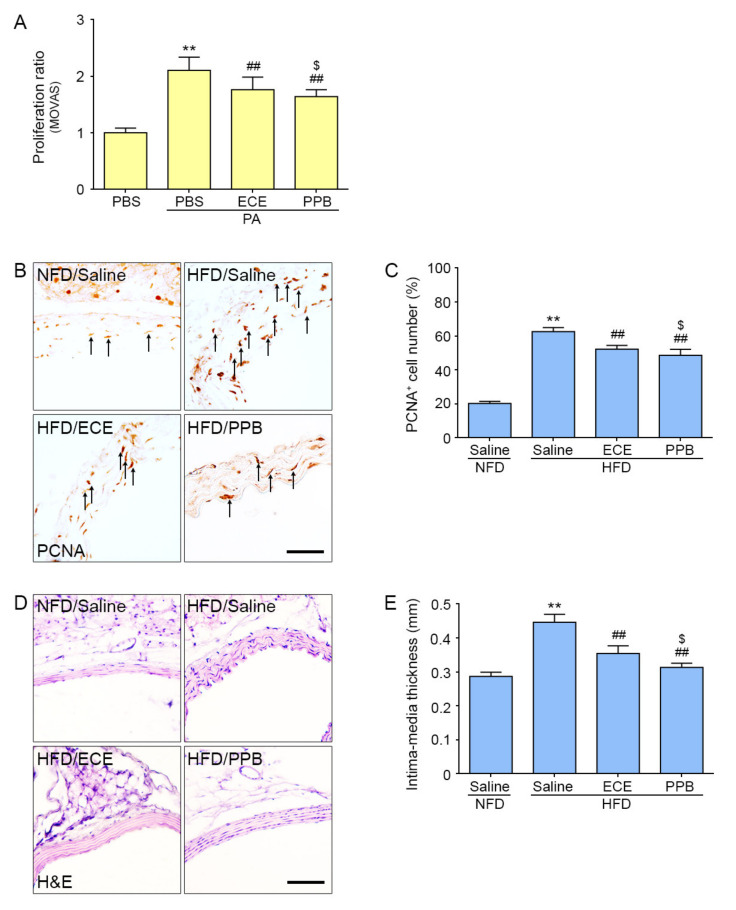
Effects of ECE and PPB on smooth muscle cell proliferation. (**A**) The proliferation ratio of MOVAS cells was measured using a WST(water-soluble tetrazolium) assay after incubation with PA in PBS, ECE, or PPB. The proliferation ratio was increased by treatment with PA and was significantly decreased by addition of ECE or PPB. PPB had a stronger effect on reducing smooth muscle cell proliferation than ECE. (**B**–**C**) PCNA staining, used to measure proliferation (brown, arrow) in aorta tissue, increased after mice were fed an HFD and decreased with the addition of ECE or PPB treatment. (**D**–**E**) The intima-media thickness measured from the H&E stained images increased when mice were fed an HFD and decreased with the addition of ECE or PPB. Data represent the mean ± SD. Scale bar, 100 µm. ** *p* < 0.01, vs. the PBS or NFD group; ## *p* < 0.01 vs. the PA or HFD group; $ *p* < 0.05 vs. PA/ECE or HFD/PPB group. ECE, *Ecklonia cava*; H&E, hematoxylin and eosin; HFD, high-fat diet group; H&E, hematoxylin and eosin; NFD, normal fat diet group; PA, palmitate acid; PBS, phosphate-buffered saline; PPB, pyrogallol-phloroglucinol-6,6-bieckol; PCNA, proliferative cell nuclear antigen.

The proliferation ratio of MOVAS cells, after incubation with PA in PBS, ECE, or PPB, was measured using a Water-soluble tetrazolium (WST) assay. The proliferation ratio of MOVAS treated with PA was increased significantly as compared to MOVAS without PA treatment. The proliferation ratio by PA treated MOVAS was significantly higher than that in PA and ECE treated MOVAS or in PA and PPB treated MOVAS. Treatment with PPB had a significantly greater effect on reducing the proliferation ratio than treatment with ECE (Figure 4A).

We evaluated proliferation of VSMC in the aorta of mice with proliferative cell nuclear antigen (PCNA), a marker of VSMC proliferation [24]. PCNA positive VSMCs was significantly increased in the aortas of mice fed an HFD as compared to those fed an NFD (Figure 4B,C). The number of PCNA-positive cells in the aortas of ECE- or PPB-treated mice fed an HFD was significantly reduced as compared to the number in the aortas of mice fed an HFD alone. Treatment with PPB resulted in a significantly greater reduction in the number of PCNA-positive cells as compared with mice treated with ECE (Figure 4B,C).

The intima-media thickness of the aorta of HFD-fed mice was significantly greater than that of mice fed an NFD (Figure 4D,E). The intima-media thickness of the aorta was significantly lower in mice on an HFD treated with ECE or PPB as compared to mice fed an HFD alone. The mice fed an HFD that were treated with PPB had significantly reduced intima-media thickness as compared to those fed an HFD and treated with ECE (Figure 4D,E).

## 3. Discussion

Arterial remodeling is defined as the process in which the arterial wall transforms from its normal physiologic state into a pathologic state after exposure to chronic stress stimuli [25]. Various processes are involved in the arterial remodeling process, including inflammation, oxidative stress, lipid accumulation, and degradation of the ECM. VSMCs are mainly engaged in arterial remodeling and induce changes in the morphology and properties of the arterial wall [25].

VSMCs exhibit cellular plasticity and lead to phenotype switching toward synthetic VSMCs, which is induced by stress stimuli. VSMC phenotype switching initiates or performs remodeling processes by increasing the synthesis of the ECM and enhancing cell proliferation, migration, and contraction [25]. Phenotype switching of VSMCs is associated with various pathologies, including the development of hypertension, atherosclerosis, aneurysm formation, intimal/medial calcification, and vascular fibrosis, which are involved in increased cardiovascular morbidity and mortality [25].

HFD-induced hyperlipidemia has been reported to induce a phenotypic switch in VSMCs from the contractile to a synthetic phenotype [26]. It has also been reported that the CCR5/CCL5 pathway is involved in the VSCM phenotype switching induced by hyperlipidemia [17]. CCL5 induces VSMC phenotype changes to synthetic one through NF-kB dependent pathway [17]. In our study, expression of CCL5/CCR5 was increased by treatment with PA in MOVAS and by HFD in mice. PPB attenuated the expression of CCL5/CCR5 in VSMCs exposed to hyperlipidemic conditions in both in vitro and in vivo models. The expression of NF-kB was increased in the aorta of HFD fed mice and it was decreased by PPB. In addition, the expression of NF-kB of PA treated MOVAS was decreased by PPB (Figure 1).

Under normal physiological conditions, VSMCs exhibit a contractile phenotype, and highly express α-SMA, SM22-α, and SM-MHC [7,27,28]. In our study, the aortas of mice fed an HFD had decreased expression of α-SMA, SM-MHC, and SM22α as compared to mice fed NFD, and expression of these factors was restored after treatment with PPB (Figure 2A–C). The expression of α-SMA, Sm-MHC, and SM22α in PA-treated MOVAS was decreased and were restored after treatment with PPB (Figure 2D–F).

Our results showed that PPB treatment reduced the phenotype switching to a synthetic phenotype in both the in vitro and in vivo models. We confirmed that CCR5 is involved in phenotype switching using siCCR5-MOVAS (Figure 2G–I). The expression of contractile markers of VSMC in PA-treated siCCR5-MOVAS did not significantly increase after treatment with PPB. Those results suggested the PPB decreased expression of CCR5, which was increased by PA, resulting in decreased phenotype switching in VSMCs.

Hyperlipidemia has been shown to induce VSMC proliferation, which leads to vascular remodeling [29,30]. The CCL5/CCR5 pathway is involved in proliferation of VSMCs due to hyperlipidemia. Our study suggests that VSMC proliferation, which was evaluated by the number of PCNA-positive cells, increased in the aortas of mice fed an HFD as compared to those of NFD mice. In the in vitro model, PA-treated MOVAS had an increased cell proliferation ratio that was reduced with the addition of PPB (Figure 4). In MOVAS, the mRNA levels of CDK2, CDK4, cyclin D1, and cyclin E, which are involved in cell cycle regulation, was significantly increased by PA. Addition of PPB significantly reduced the levels of the cell cycle regulation factors, resulting in decreased proliferation of VSMCs (Figure 3). VSMC proliferation leads to vascular remodeling, such as increasing thickness of vascular wall. In our study, the intima-media thickness of the aorta dute to HFD was attenuated by treatment with PPB. These results suggest that PPB reduces VSMC proliferation and decreases the intima-media thickness of aortas induced by HFD.

Based on the evidences that CCL5/CCR5 pathway involves in progression of atherosclerosis or intimal hyperplasia, several studies reported that maraviroc, an antagonist of CCR5, showed decreasing atherosclerosis or intimal hyperplasia [31,32]. Maraviroc is a drug for human immunodeficiency virus type 1 (HIV-1) infection and it shows effect of inhibiting HIV-1 entry by blocking CCR5 on the CD4+ T-cells [33]). Maraviroc is known that has possible side effect of hepatotoxicity and association with malignancies [34]. Thus, further studies are needed to use maraviroc for decreasing atherosclerosis or intimal hyperplasia.In our study, PPB induced downregulation of CCL5/CCR5 pathway. PPB is a natural compound from edible alga, hence it might be much safer and cheaper than synthetic compound, such as maraviroc.

In conclusion, our study showed that PPB reduced the upregulation of CCL5/CCR5 due to hyperlipidemia and decreased VSMC proliferation and phenotype switching which may accelerate vascular remodeling. PPB may be a potential drug to treat atherosclerosis or intimal hyperplasia through modulating VSMC proliferation and phenotype switching.

## 4. Materials and Methods

### 4.1. Ecklonia Cava Extraction (ECE) and Pyrogallol-Phloroglucinol-6,6-bieckol (PPB) Preparation

ECE and PPB preparation methods were performed as described previously [35].

### 4.2. Diet Induced Obesity (DIO) Animal Model

All animal experiments were reviewed by the Ethical Principles in Institutional Animal Care and Use Committee of Gachon University (approval number; LCDI-2017-0034).

Male C57BL/6N mice (8 weeks of age) were obtained from Orient Bio (Seongnam, Korea) and kept at a constant temperature of roughly 23 °C, relative humidity of 50%, and a light cycle of 12 h dark and12 h light. Mice were fed different diets as described below and provided drinking water *ad libitum* for eight weeks. For the first four weeks, mice received either a regular normal fat diet (NFD) or a 45% HFD (Research Diet, USA) adapted from a previous study [36].

For the last four weeks, HFD mice were orally administered either 0.9% normal saline, ECE (HFD/ECE; 100 mg/kg/day), or PPB (HFD/PPB; 2.5 mg/kg/day), along with either a regular normal diet or high-fat diet. The doses of ECE and PPB used here were the same as those used in a previous study [36]. At the end of the eight-week study period, all mice were sacrificed and harvested for the aortic tissue and blood.

### 4.3. Cell Culture and Experimental Cell Models

#### 4.3.1. VSMC Culture and Experimental Cell Model

VSMCs (MOVAS) were purchased from the American Type Culture Collection. These cells were cultured in high-glucose dulbecco’s modified eagle’s medium supplemented with 10% fetal bovine serum and antibiotic G-418 at 37 °C with 5% CO_2_.

VSMCs were treated with 200 μM of palmitate for 24 h, as described previously [20].

#### 4.3.2. Treatment with ECE and PPB

VSMCs were treated with ECE (50 µg/mL) and PPB (1.8 µg/mL) for 24 h, as described previously [20,37].

#### 4.3.3. Small Interfering RNA (siRNA, CCR5) Transfection

MOVAS cells were cultured in 60 mm^2^ dishes to 70–80% confluencey. The cells were then transfected with 100 nmol/L of CCR5 siRNA (Thermo Fisher Scientific, Boston, MA, USA), using lipofectamine reagent.

### 4.4. VSMCs Proliferation Ratio Measurement

MOVAS cells were cultivated in a 96-well plate (5 × 10^3^ cells//well) for 24 h and washed with phosphate-buffered saline (PBS). WST solution was added (100 μL/well) for 4 h at 37 °C. Absorbance was measured at 570 nm using a VERSAmax tunable microplate reader (Molecular Devices, CA, USA).

### 4.5. RNA Extraction and Complementary Deoxyribonucleic Acid (cDNA) Synthesis

Total RNA was extracted using RNAiso Plus (TAKARA, Tokyo, Japan), according to the manufacturer’s instructions. RNAiso Plus (0.5 mL) was mixed with chloroform (0.1 mL) and incubated at room temperature for 7 min. The mixture was centrifuged for 15 min at 4 °C at 12,000× *g*. The supernatant was collected in a new tube, mixed with 0.25 mL of 100% isopropanol, gently shaken, and then centrifuged again to precipitate the RNA. The supernatant was discarded, and the RNA pellet was washed with 70% ethanol and centrifuged at 7500× *g* for 5 min at 4 °C. The dried pellet was dissolved in 30 μL of diethyl pyrocarbonate water and RNA was quantified using Nano Drop 2000 (Thermo Fisher Scientific, Boston, MA, USA). Total RNA (1 µg) was used to synthesize cDNA using the cDNA synthesis kit.

### 4.6. Real-Time Reverse Transcription Polymerase Chain Reaction (qRT-PCR)

qRT-PCR was performed to assess mRNA levels. The reaction mixtures were prepared in wells of 384-well plates and contained 0.8 µL 10 pM primer (Table S1), 1 µg cDNA template (2 µL), and 5 µL SYBR Green. Analysis was performed using CFX384 Touch (Bio-Rad, Hercules, CA, USA).

### 4.7. Triglyceride Measurment Assay

To measure triglyceride levels in the serum, we obtained blood in an ethylenediaminetetraacetic acid (EDTA) buffer-coated tube (300–500 μL). Tubes were centrifuged at 2000 rpm for 20 min at room temperature. After the collection of the supernatant from the tube, total triglyceride levels were measured by KPNT company (Cheongju, Gyeonggi-do, Korea) following the peroxidase-coupled method [38]. Optical density was validated using a microplate reader (Molecular Devise, San Jose, CA, USA) at 510 nm wavelength.

### 4.8. Total Cholesterol Measurment Assay

To measure the total cholesterol levels in the serum, we obtained blood in an EDTA buffer-coated tube (300–500 μL). Tubes were centrifuged at 2000 rpm for 20 min at room temperature. After the collection of the supernatant from the tube, total cholesterol levels were measured by KPNT (Cheongju, Gyeonggi-do, Korea) following the cholesterol oxidase/peroxidase (CHOD-POD) method [39]. Optical density was validated by microplate reader at 510 nm wavelength (Molecular Devise, San Jose, CA, USA).

### 4.9. Immunohistochemistry (3, 3 –diaminobenzidine; DAB)

Blocks of paraffin-embedded aorta tissue were sectioned to a thickness of 7 µm, placed on a coating slide, and dried at 40 °C for 24 h. Slides were deparaffinized and incubated in 0.3% H_2_O_2_ for 30 min. Then, slides were rinsed three times with PBS and incubated in normal animal serum to block non-specific binding, incubated with anti-PCNA (Abcam, Cambridge, UK; at a 1:200) dilution at 4 °C, followed by three additional rinses with PBS. Slides were then treated with biotinylated secondary antibodies from the ABC (Avidin/Biotin Complex) kit (at a 1:200 dilution), incubated for 1 h with blocking solution, and rinsed three times with PBS. Slides were left to react with 3,3′-diaminobenzidine (DAB) substrate for 15 min and were subsequently mounted with a coverslip and DPX mounting solution (Dibutylphthalate Polystyrene Xylene). Images were detected using a light microscope (Olympus, Tokyo, Japan), and quantification of the intensity of the brown color was performed using Image J software (NIH, Bethesda, MD, USA).

### 4.10. Histological Hematoxylin and Eosin (H & E) Staining

Blocks of paraffin-embedded aorta tissue were sectioned at a thickness of 7 µm, placed on a slide, and dried at 40 °C for 24 h. Slides were deparaffinized and incubated in hematoxylin for 1 min, eosin for 20 s, followed by three rinses in PBS. Finally, slides were mounted with a coverslip and DPX mounting solution (Dibutylphthalate Polystyrene Xylene), followed by detection with a light microscope. The media-intima thickness was measured using Image J software (NIH, Bethesda, MD, USA).

### 4.11. Statistical Analysis

Statistical differences among 3 or 4 groups were determined using the non-parametric Kruskal-Wallis test, and differences between two groups were compared using the Mann–Whitney U post-test. Experiments were performed in triplicate, and results were presented as means ± SD. The analysis was conducted using SPSS version 22 (IBM Co., New York, NY, USA).

## Supplementary Materials

The following are available online at https://www.mdpi.com/1660-3397/18/8/393/s1, Table S1: List of primers used for quantitative real-time polymerase chain reaction; Figure S1: CCR5 silencing after treatment with ECE and PPB.
