# Supplementary material for: Pyrogallol-Phloroglucinol-6,6-Bieckolon Attenuates Vascular Smooth Muscle Cell Proliferation and Phenotype Switching in Hyperlipidemia through Modulation of Chemokine Receptor 5
Source: Mar Drugs. 2020 Jul 27;18(8):393. doi: 10.3390/md18080393 (PMC7460451; doi:10.3390/md18080393)
Supplement: Supplementary file 1 [file marinedrugs-18-00393-s001.pdf]

marine drugs

MDPI

Supplementary Tables

Pyrogallol-Phloroglucinol-6,6-Bieckolon Attenuates Vascular Smooth Muscle Cell Proliferation and Phenotype Switching in Hyperlipidemia Through Modulation of Chemokine Receptor 5

Seyeon Oh [1,†], Myeongjoo Son [1,2,†], Chul-Hyun Park [3], Ji Tae Jang [4], Kuk Hui Son [3,*] and Kyunghee Byun [1,2,*]

[1] Functional Cellular Networks Laboratory, Lee Gil Ya Cancer and Diabetes Institute, Gachon University, Incheon 21999, Korea; seyeon8965@gmail.com (S.O.), mjson@gachon.ac.kr (M.S.)
[2] Department of Anatomy & Cell Biology, Graduate School of Medicine, Gachon University, Incheon 21936, Korea
[3] Department of Thoracic and Cardiovascular Surgery, Gachon University Gil Medical Center, Gachon University, Incheon, Korea; cdgpch@gilhospital.com
[4] Aqua Green Technology Co., Ltd., Smart Bldg., Jeju Science Park, Cheomdan-ro, Jeju 63243, Korea; whiteyasi@gmail.com
[*] Correspondence: dr632@gilhospital.com (K.H.S.); khbyun1@gachon.ac.kr (K.B.); Tel.: +82-32-899-6511 (K.H.S. and K.B.)
[†] These authors contributed equally to this work.

Table 1. List of primer for quantitative real-time polymerase chain reaction.

Gene		Primers
**β-actin**	Forward	5'-ACA AAG CTG TTC AGT GTC TCC A-3′
	Reverse	5'-CTC CGT TTC CAG AAT ACA CAC A-3′
α-SMA	Forward	5′-TGA CGC TGA AGT ATC CGA TAG-3′
	Reverse	5′-AAG AGT GGT GCC AGA TCT TTT C-3′
CCR5	Forward	5′-ACA CTA CCA TTC TGG GCT CAC T-3′
	Reverse	5′-CAA TGT GAT AGA GCC CTG TGA A-3′
CCL5	Forward	5′-CTC CAA TCT TGC AGT CGT GTT-3′
	Reverse	5′-CTT GAA CCC ACT TCT TCT CTG G-3′
CDK2	Forward	5′-GTG GTG TAC AAA GCC AAA AAC A-3′
	Reverse	5′-CAC CTT CAG TCT CAG TGT CGA G-3′
CDK4	Forward	5′-ATG TCT GTG CTA CTT CCC GAA C-3′
	Reverse	5′-CCT CAG GTC CTG GTC TAT ATG C-3′
CyclinD1	Forward	5′-AAT GGA ACT GCT TCT GGT GAA C-3′
	Reverse	5′-AGG AAG TGT TCG ATG AAA TCG T-3′
CyclinE	Forward	5′-AAT GGA GGT GTG CGA AGT CTA T-3′
	Reverse	5′-GAT GCC ATG TAA CGA TCA AAG A-3′
NF-κB	Forward	5'-AGA AAT CCT ACC CAC AGG TCA A-3′

	Reverse	5'-CAT TTG TGA CCA ACT GAA CGA T-3′
SM22α	Forward	5′-AGA ACT TCC AGA AGT GGC TCA A-3′
	Reverse	5′-GAG GCC TGG ATC TTC TTT ACT G-3′
SM-MHC	Forward	5′-CGG GAA GAC AGA AAA TAC CAA G-3′
	Reverse	5′-ATG GTC CTT TCT TCC TTT GTG A-3′
